# Genome-wide assays that identify and quantify modified cytosines in human disease studies

**DOI:** 10.1186/1756-8935-8-5

**Published:** 2015-01-22

**Authors:** Netha Ulahannan, John M Greally

**Affiliations:** Department of Genetics, Albert Einstein College of Medicine, Center for Epigenomics and Division of Computational Genetics, 1301 Morris Park Avenue, Bronx, NY 10461 USA

**Keywords:** DNA methylation, 5-methylcytosine, Epigenomic, Assay, CpG island, Enhancer, microarray

## Abstract

The number of different assays that has been published to study DNA methylation is extensive, complemented by recently described assays that test modifications of cytosine other than the most abundant 5-methylcytosine (5mC) variant. In this review, we describe the considerations involved in choosing how to study 5mC throughout the genome, with an emphasis on the common application of testing for epigenetic dysregulation in human disease. While microarray studies of 5mC continue to be commonly used, these lack the additional qualitative information from sequencing-based approaches that is increasingly recognized to be valuable. When we test the representation of functional elements in the human genome by several current assay types, we find that no survey approach interrogates anything more than a small minority of the nonpromoter *cis*-regulatory sites where DNA methylation variability is now appreciated to influence gene expression and to be associated with human disease. However, whole-genome bisulphite sequencing (WGBS) adds a substantial representation of loci at which DNA methylation changes are unlikely to be occurring with transcriptional consequences. Our assessment is that the most effective approach to DNA methylation studies in human diseases is to use targeted bisulphite sequencing of the *cis*-regulatory loci in a cell type of interest, using a capture-based or comparable system, and that no single design of a survey approach will be suitable for all cell types.

## Introduction

While it is customary to think of DNA as containing four nucleotides - adenine, thymine, guanine and cytosine - the cytosines in many organisms represent targets for several covalent modifications, now recognized to include 5-methylcytosine (5mC), 5-hydroxymethylcytosine (5hmC), 5-carboxylcytosine (5caC) and 5-formylcytosine (5fC) (reviewed in [1]). Of these, 5mC is the most abundant alternative version of cytosine [2]. The cytosine 5mC was first recognized as a toxic extract from *Mycobacterium tuberculi* in 1898 and named tuberculinic acid as a result [3]. Studies of neoplastic cells in the 1980s revealed differences in 5mC content compared with nontransformed cells [4, 5], opening up the possibility that studies of human development and diseases, including cancer in particular, may involve this nucleotide variant [6].

The decades since have seen a steady progression in our capability to study 5mC more broadly throughout the genome, at increasing resolution and in an expanding range of organisms. Some of the earliest approaches involved performing Southern blots using DNA pre-digested with restriction enzymes that are sensitive to the presence of 5mC [7]. This approach allowed some of the earliest observations of cancer-related 5mC changes [4] and revealed the role of 5mC in developmental regulation of gene expression due to genomic imprinting in mammals [8]. The development of the polymerase chain reaction (PCR) led to new assays being designed, with some based on ligation-mediated PCR [9] and others on the amplification across the sites that could be digested by a specific restriction enzyme [10]. The latter type of assay enabled the sensitive detection of the presence of methylated DNA at loci where 5mC was normally completely absent, which became a major means of testing for the presence of abnormal DNA methylation in cancer in particular [11, 12].

A technical breakthrough in the technology to measure DNA methylation was the development of bisulphite conversion, which was found to deaminate selectively cytosines but not 5mC [13]. Once converted, downstream assays could be applied, including not only restriction enzyme digestion but also currently-available sequencing-based approaches. The restriction enzyme-based approaches included COBRA (COmbined Bisulphite Restriction Analysis [14]), which generally exploited the destruction by bisulphite exposure of a pre-existing restriction enzyme site or the creation of a new one. However, for the first time, DNA sequencing could be applied to the product of the bisulphite treatment, generally involving PCR of the bisulphite-treated DNA followed by sequencing [13]. This generates nucleotide-resolution quantification of DNA methylation, while cloning and sequencing of the PCR product add allelic information, shedding further light upon processes like genomic imprinting [15]. Other technologies were also applied downstream of bisulphite treatment, including pyrosequencing [16] and mass spectrometry [17], which were designed to enable more accurate quantification of 5mC at sites within the amplicons tested.

**Figure 1:**
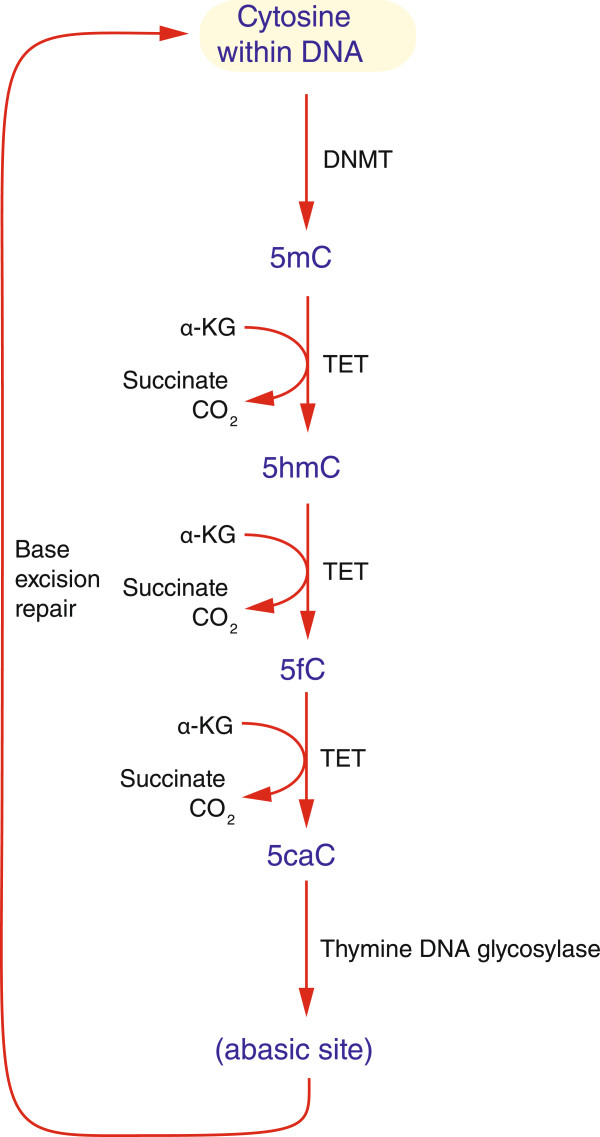
**Cytosine variants and their production.** We show how cytosine within DNA can be acted upon by DNA methyltransferases (DNMT) to generate 5-methylcytosine (5mC), which can subsequently be oxidized by TET enzymes through the 5-hydroxymethylation (5hmC), 5-formylcytosine (5fC) and 5-carboxylcytosine (5caC) variants, favoring the activity of thymine DNA glycosylase to create an abasic site that can then be repaired to add back an unmethylated cytosine to complement the guanine on the other strand. As well as alpha-ketoglutarate (α-KG), another known TET enzyme cofactor is ascorbic acid (vitamin C).

**Figure 2:**
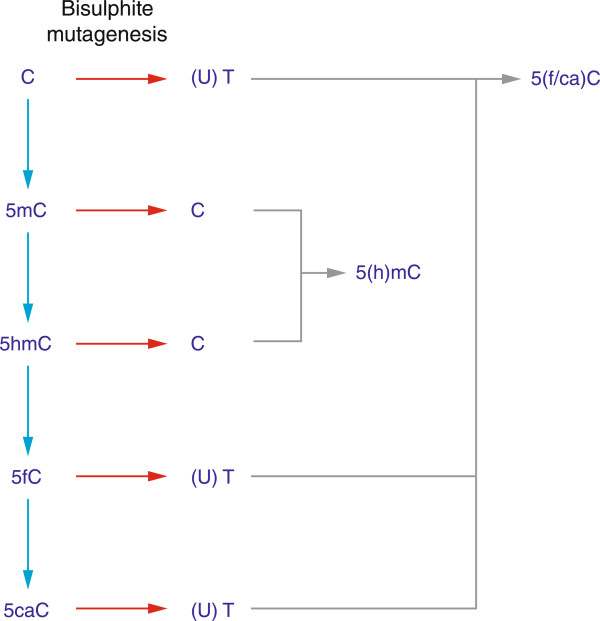
**The effects of bisulphite conversion on different cytosine variants.** Bisulphite conversion deaminates not only unmodified cytosine but also 5fC and 5caC [27] to uracil, which is then amplified and sequenced as a thymine. Both 5mC and 5hmC are resistant to bisulphite conversion and are sequenced as cytosines. While the output of bisulphite sequencing has been generally described to be the ratio of 5mC/(5mC + C), more correctly it should be described as (5mC + 5hmC)/(5mC + 5hmC + 5fC + 5caC + C), which only approximates 5mC/(5mC + C) when the other cytosine variants are in very low proportions.

The development of massively-parallel sequencing (MPS) in the last decade has allowed the product of bisulphite conversion to be sequenced on a scale never previously possible. During the MPS era, it has emerged that 5mC is not the only cytosine variant in the genome, but is accompanied by lower proportions of 5hmC [2], 5caC and 5fC [18] (Figure 1). It became apparent that previous assays involving bisulphite conversion read each of these cytosine modifications differently [19] (Figure 2), which prompted the need to re-evaluate prior assumptions about distributions of modified cytosines in the genome. Assay development for these new modifications is focused on exploiting MPS technologies, resulting in some intriguing early observations about the distributions of some of these cytosine variants. For example, 5hmC can be tested using Tet-assisted bisulphite sequencing (TAB-seq [20]) or oxidative bisulphite sequencing (oxBS-seq [21]), with chemical modification-assisted bisulphite sequencing (CAB-seq) developed for 5caC [22], and reduced bisulphite sequencing (redBS-seq) for 5foC [23]. Within the genome of mouse embryonic stem (ES) cells, 5hmC has been found to be enriched at promoters, especially those encoding bivalent chromatin domains and exons [24]. CpG islands in mouse ES cells appear to be especially enriched for 5fC [25], but these studies used an affinity-based assay, which may preferentially target such CG-rich loci [26]. Definitive nucleotide-resolution mapping studies will undoubtedly be published in the near future, giving us insights into the potential function of these cytosine modifications.

There have been numerous excellent reviews that have described the panoply of DNA methylation assays currently available for use [19, 28–31]. In this review, the goal is to build upon this prior foundation with a focus on the use of MPS technologies, especially as applied to studying human diseases, including attention to the study of cytosine variants other than 5mC, and incorporating a discussion about how new insights into genomic physiology help to direct our experimental approaches to studying cytosine variants in the genome.

## Review

### Cytosine variants

To use the common terminology for mediators of epigenetic regulation, cytosine variants are established by writers, bound to by readers, and removed by erasers. Cytosine is the target of modifications in mammalian genomes, the most common modification being 5-methylcytosine (5mC), usually at a cytosine followed by a guanine, creating the CG (CpG) dinucleotide. The CG dinucleotide combination is the shortest sequence at which the opposite strand can have a complementary cytosine within the dinucleotide, creating a palindromic sequence. This allows a symmetrical modification of the cytosines on both strands, which becomes important during cell division when both of the daughter chromatids form a hemimethylated state. The newly-synthesized DNA has an unmodified cytosine that complements the template strand with the 5mC. The recognition of this hemimethylated state and the subsequent enzymatic restoration of symmetrical 5mC on both strands represent the best-characterized molecular mechanisms for heritability of nongenetic information. As such, 5mC represents the one clearly epigenetic process in mammalian genomes. The DNA methyltransferase enzyme involved in restoring symmetrical DNA methylation following replication is DNMT1 [32], whereas the DNMT3A and DNMT3B enzymes can act upon nonreplicating DNA to induce DNA methylation *de novo* [33]. The DNMTs thus represent the writers of the 5mC state, with different members of this protein family acting at different stages of the cell cycle.

5-methylcytosine is now just one of several cytosine modifications recognized to occur in mammalian DNA. The relative abundance of each of the other variants is substantially less, delaying their recognition and the development of assays for their analysis. 5mC can be oxidized by the TET family of enzymes to form 5-hydroxymethylcytosine (5hmC), which is further oxidized by TETs to 5-formylcytosine (5fC) and to 5-carboxylcytosine (5caC) [34]. These are believed to be steps involved in the active transition from 5mC to unmodified cytosine. The TET enzymes can thus be thought of as erasers of the 5mC state, but also as writers of the 5hmC, 5fC and 5caC states (Figure 1).

The readers of 5mC are characterized by proteins with methyl-binding domains (MBDs) and include the MECP2 protein, which, when mutated, has been associated with the development of Rett syndrome [35] and autism spectrum disorder [36]. A relative preference for 5hmC and 5fC compared to cytosine or with 5mC has been identified for several DNA-binding proteins [37], suggesting that these modifications are not merely transient stages in demethylation but have functional relevance in transcriptional control. Some of the readers of 5mC have been tested for their relative capacity to bind to 5hmC, with the MBD of MECP2 showing a strong preference for 5mC over 5hmC [38]. The presence of 5mC at certain sites inhibits the ability of certain transcription factors and DNA-binding proteins to bind to their cognate motifs [39], so the absence of 5mC could, paradoxically, be said to have its own readers. When 5mC is found at a *cis*-regulatory element, it has usually been found to be associated with the silencing of the associated gene [40], leading to the cytosine modification being described as repressive. As the majority of DNA in mammalian genomes is 5mC-modified [41], and *cis*-regulatory sites are frequently characterized by their lack of 5mC [42–45], the importance of 5mC may be mostly in terms of where it is not located in the genome.

Pericentromeric regions of repetitive DNA are highly enriched for 5mC through the action of DNMT3B [46]. Between this localization to cytogenetically heterochromatic regions and the silencing effect of 5mC at *cis*-regulatory loci, the assumption has been long held that more methylated cytosines define the more heterochromatic regions of the genome. We have described the paradoxical finding that the majority of the human genome is characterized by enrichment of 5mC in the vicinity of regions with higher levels of transcription, earlier DNA replication timing and generally increased DNase hypersensitivity [47]. This targeting of 5mC to regions of euchromatin may be at least in part attributable to the effect of transcription, which induces the local accumulation of 5mC [48, 49].

A consequence of 5mC is increased propensity to mutations. When an unmodified cytosine undergoes spontaneous deamination, it becomes a uracil, which is readily recognized not to be part of the native DNA sequence and is efficiently removed and repaired. The deamination of 5mC, on the other hand, generates thymine, which could be native to the DNA sequence. The thymine in the double-stranded DNA is mismatched with a guanine downstream from a 5mC on the other strand, creating a T:G mismatch and hemimethylated DNA that is specifically recognized by MBD4 [50], a thymine DNA glycosylase that removes the thymine for replacement by cytosine. This recognition and replacement must not be wholly effective, as CGs dinucleotides are hotspots of DNA mutations in somatic cells [51], and CGs are extremely depleted in genomes of organisms that have 5mC [52, 53]. The depletion of CGs is not uniform in the genome, with a subset of the genome remaining relatively CG-dense. These regions of CG density have been described as CpG islands [54] and have been the target of many studies of DNA methylation, prompted by the observation that in certain cancers these normally unmethylated loci can become the target for acquisition of 5mC, which is referred to as the CpG Island methylator phenotype (CIMP) [55]. The presence of 5mC at these loci in nonmalignant cells is a rare event, including a small subset of canonical gene promoters [56] as well as loci undergoing X chromosome inactivation [57] and genomic imprinting [58]. CpG islands are frequently located at gene promoters and tend to be unmethylated in noncancerous cells whether the associated gene is active or inactive [59], generally providing little predictive information about gene expression in primary, non-neoplastic cells. The CpG island annotation is surprisingly simplistic, based on the observed to expected ratio of CG dinucleotides and the (G + C) mononucleotide proportion in windows of ≥200 bp [54]. When such sequences are annotated in the human genome, approximately 350,000 loci match these criteria, with 92% located within repetitive elements. Those used for public genome browser annotations start by removing the repetitive sequences, focusing on the approximately 28,000 located within unique sequence [60]. If the base composition characteristics of CpG islands are functionally important, it is difficult to rationalize why the removal of 92% of loci with such characteristics is warranted. With the alternative hypothesis that the generally unmethylated status of CpG islands is the more valuable annotation, an innovative approach was employed that used the CXXC protein domain, known to bind selectively to unmethylated CG dinucleotides [61], to pull down ‘nonmethylated islands’ (NMIs) from DNA of multiple species. The authors describe finding that while NMIs tend to be enriched for the base compositional characteristics of CpG islands, the property of being unmethylated is better than the CpG island annotation at predicting regulatory elements in a genome and is more conserved across genomes of different species [62].

Our tradition of directing 5mC assays towards the analysis of the CpG islands annotated in genome browsers is therefore likely to be suboptimally informative when seeking to define loci where changes in 5mC are associated with transcriptional changes. In fact, 5mC changes flanking the CpG island itself have been found to be more correlated with nearby gene transcriptional levels than 5mC variability within the CpG island itself. This so-called CpG island shore represents the 2 kb region flanking the CpG island and shows increased DNA methylation associated with decreased gene expression levels [63]. An insight into these CpG island shores comes from our recent study of human CD34+ hematopoietic stem and progenitor cells (HSPCs), which reveal these shores to be highly enriched for sequences with the chromatin characteristics of enhancers [64]. In now appears from examples in cancer [65], other human diseases [66–70] and normal cells [42, 71], that DNA methylation variability at distal *cis*-regulatory loci such as enhancers is more correlated with gene expression than DNA methylation at promoters or CpG islands. *Cis*-regulatory loci now appear to represent the most rewarding potential sites for 5mC assays.

The reason for 5mC to exist in broad regions of the mammalian genome, avoiding *cis*-regulatory elements, has been the subject of speculation. It has been proposed that 5mC exists to repress transposable elements (the host defense hypothesis [72]), or to prevent activity of cryptic promoters (transcriptional noise hypothesis [73]), and that it is protective against chromosomal instability [74]. Oddly, the times during mammalian development when transposon activation or chromosomal rearrangements could be most damaging probably include during gametogenesis and early development, both characterized by profound demethylation throughout the genome [75], with evidence for activation of transposable elements [76]. There is some published evidence to support the transcriptional noise hypothesis [77]. The chromosomal instability hypothesis is mostly supported indirectly by cytogenetic findings in DNMT3B deficiency (ICF syndrome [46]), the increased rate of loss of heterozygosity in tumors formed in mice with *Dnmt1* mutations [78] and the induction of fragile sites [79] and chromosome breakage [80] by DNMT1 inhibitors. The chromosome breakage may, however, be attributable to adducts between the drug and DNMT1 and not the hypomethylation of the DNA itself [81]. Overall, even after decades of studies of 5mC physiology, the necessity for it to be located throughout the mammalian genome remains incompletely understood.

### Current approaches to studying DNA methylation changes in human diseases

The first insights into the potential role of DNA methylation alterations in human diseases were from cancer studies at individual loci, as described earlier. The extreme changes of DNA methylation in cancer, with global shifts and the unusual acquisition of DNA methylation at CpG island promoters, established a paradigm for the study of other diseases. The range of human diseases and phenotypes being studied is now extremely broad, including aging [82], immunological [83], renal [66], neurological [84], pulmonary [85], gastrointestinal [86], infectious [87] and other diseases. The same extreme changes are rarely seen in these nonmalignant conditions, with the exception of certain viral infections [88]. Another common finding is that the loci that change DNA methylation almost never do so in a way that involves switching between extreme hypomethylation to extreme methylation. Instead, the changes seem to be intermediate in degree with values as low as a few percent distinguishing the groups.

The observation of small changes in methylation between groups has three practical implications. First, it indicates that the changes in DNA methylation occur in a subset of the pool of cells sampled. An individual cell cannot have an intermediate DNA methylation value such as 20%: either both cytosines are methylated on the homologous chromosomes (100%), neither is methylated (0%), or in certain situations there is 50% DNA methylation when one but not the other allele is methylated. A change of DNA methylation from 20% to 40% between samples means that there has to be a mosaic subpopulation of alleles or cells that changes its proportion. This raises the question of whether the effects are purely due to differences in cell subcomposition between the samples. This has retrospectively been found to be the case in a number of studies of aging using peripheral blood leukocytes [89]. Re-analysis of these samples, using DNA methylation patterns known to characterize individual leukocyte subtypes to deconvolve the global patterns observed, revealed that the effects of aging were almost wholly attributable to cell subtype composition, and may not reflect epigenetic changes in any of the cells studied [89].

The second consequence of intermediate DNA methylation changes occurring in human disease studies is that it puts pressure on the genome-wide assay to be capable of detecting small changes. It has been proposed that the kind of deconvolution approach used to understand the effects of cell subcomposition in aging [89] can be applied analytically to remove this confounding effect and allow genuine epigenetic changes to be detected [90]. This is a reasonable assumption and has potential to rescue studies that are based on the use of mixed cell types. What remains problematic is the possibility that the epigenetic changes may only be occurring in a proportion of a subtype of the cells studied. Even purified cells have been found to show intermediate changes in DNA methylation in these kinds of studies [91–93], raising the possibility that if a cell subtype undergoing the epigenetic change represents, for example, 10% of the mixed population of cells being studied, and the DNA methylation changes associated with the disease are in the range of 20% in that affected cell subtype but no other cells in the population change their DNA methylation, the genome-wide assay has to detect (0.10 × 0.20) a 2% change in DNA methylation in the mixed cell population. This is a problem if the genome-wide assay does not have sufficient resolution or sensitivity to detect changes of such limited magnitude.

The third problem is not immediately intuitive, and represents an indirect implication of mosaic epigenetic events. A question that frequently arises is whether chromatin immunoprecipitation (ChIP)-based studies can be used in human diseases, instead of the common approach of studying DNA methylation. ChIP followed by MPS (ChIP-seq [94]) is the genome-wide assay that allows us to map chromatin components such as post-translational histone modifications, DNA-binding proteins and chromatin structure. The assay results in a binary peak/no peak output, and while there has been some progress making the assay more quantitative [95], it is not yet an assay that allows the distinction between, for example, a sample in which 20% of the cells have trimethylation of lysine 4 in histone H3 (H3K4me3) and another sample in which the proportion of cells with H3K4me3 is 40%. More development of ChIP-seq is needed before it will be capable of detecting the epigenetic changes that appear to characterize human diseases. While it would be of immense value to be able to add studies of other transcriptional regulatory processes in human diseases using quantitative ChIP-seq, at present our focus in human disease studies remains limited to the study of DNA methylation.

### Global DNA methylation assays

A first step in many human disease studies of DNA methylation is the quantification of global DNA methylation levels, prompted by the observed global shifts in DNA methylation that characterize certain tumors [96] and have also been found to occur in certain viral infections [88]. Highly quantitative tests for all cytosines in the genome include mass spectrometry [97] and high-performance liquid chromatography [2], which would also generate information about cytosine variants other than 5mC, but which both require specialized equipment and expertise that are not universally available. Pyrosequencing [98] also requires specific equipment and has been used to quantify the C/T ratio in bisulphite-converted DNA to measure DNA methylation at specific loci [99]. By targeting the highly repetitive L1 LINE (LINE1) and Alu SINE sequences, an estimate of global DNA methylation can be acquired. It should be noted that this represents types of sequences in the genome that are normally highly methylated [100], so the test is more sensitive to a global loss of DNA methylation than its global acquisition. The luminometric methylation assay (LUMA) also uses pyrosequencing but is preceded by restriction enzyme (RE) digestion of the genomic DNA, measuring the quantity of overhanging ends of fragments digested by methylation-sensitive REs (for example, HpaII), normalized to digestion by a methylation-insensitive isoschizomer (for example, MspI) and controls for restriction enzyme digestion (for example, EcoRI) [101]. HpaII and MspI represent about 8% of CGs in the genome [102] and are present in both methylated and unmethylated contexts, allowing LUMA to report global shifts in DNA methylation that can also be from less to more methylated states. An excellent recent review compares these global DNA methylation quantification approaches [103], with the development of a new mass spectroscopy-based isotope tracing technique reported more recently [104] that demonstrates the additional ability to test the dynamics of these modified cytosines in living cells.

### From microarrays to massively parallel sequencing

Of the assays used for genome-wide DNA methylation studies in human diseases, microarrays currently appear to be the most widely used [105]. This reflects some of the pragmatic choices that have to be made by an investigator when choosing how to perform a maximally informative study of a human disease phenotype. We have described earlier why DNA methylation represents a better choice at present than ChIP-seq for epigenomic and transcriptional regulatory studies; the choice within DNA methylation assays then bifurcates into a microarray or MPS-based approach. It should be noted that microarrays and MPS merely map and quantify the results after a pre-treatment of the DNA. This pre-treatment can be affinity-based, selective enriching methylated [106] or unmethylated [107] DNA. The pretreatment can also be based on the use of methylation-sensitive restriction enzymes [108] or bisulphite conversion of DNA [109]. Of these, the commonly-accepted gold standard approach is shotgun whole-genome bisulphite sequencing (WGBS), which generates nucleotide-resolution, quantitative information at most cytosines throughout the genome. It remains, however, a very expensive assay to perform, prompting the development of what could be called ‘survey’ assays that test a subset of the cytosines dispersed throughout the genome. The oligonucleotides on microarrays have traditionally been designed to represent the sites believed by researchers to be informative locations for DNA methylation changes, usually enriching representations at annotated promoters, CpG islands and their shores, and loci such as imprinted differentially-methylated regions (DMRs) [110, 111]. Survey approaches using MPS divide into two groups, the reduced representation bisulphite sequencing (RRBS) approach that uses a size range of fragments generated by RE digestion to target deep sequencing to these specific loci [111], and assays that use methylation-sensitive RE digestion to generate tags at these sites, proportionally representing the degree of DNA methylation at those sites, exemplified by our HELP-tagging assay [108].

It has been shown that both MPS and microarray-based data correlate well with WGBS [112, 113]. Approaches involving MPS generate information not possible from microarrays, including SNP detection [108, 114] and DNA methylation entropy [115, 116]. Microarrays are generally designed with the assumption that 5mC occurs only in the context of CG dinucleotides, but in certain human cell types, there are detectable levels of CHG and CHH methylation [41], which would either not be detected or would introduce unexpected effects, for example interfering with digestion by the normally DNA methylation-insensitive MspI [117]. As mentioned earlier, CG dinucleotides are very mutable and polymorphic, which is a problem for microarray designs that include a substantial component of CGs that represent known common SNPs [118, 119]. The substantial advantages to MPS in general and bisulphite sequencing in particular are becoming increasingly apparent.

It should however be recognized that bisulphite sequencing represents different cytosine modifications in distinctive ways (Figure 2). The unconverted cytosine output of bisulphite sequencing should not be taken to represent merely 5mC but also the contribution of 5hmC at that site. To resolve the relative proportions of each, both a bisulphite (5(h)mC) and a specific 5hmC assay need to be performed in parallel. Both 5fC and 5caC are read by bisulphite conversion as unmodified cytosines, requiring their specific detection using further specialized assays. The range of assays available to detect multiple cytosine variants was extensively reviewed recently [19]. The low relative proportions of 5hmC, 5fC and 5caC make their quantification by sequencing even more challenging than for 5mC, requiring a substantially deeper representation of the genome to detect alleles occurring at low frequencies. This increases the relative costs associated with these assays.

All of the prior discussion of MPS has implied the use of technologies based upon sequencing by synthesis, exemplified by the Illumina platform, indirectly measuring cytosine modifications following their chemical conversion and PCR amplification. It should, however, be noted that there are MPS platforms that use the primary DNA sequence rather than amplified derivative material, and obtain qualitative data about the sequence that appear to reflect DNA methylation, for example, the SMRT sequencing approach from Pacific Biosciences [120]. The throughput of this platform currently precludes it being able to study genomes the sizes of those of mammals, but SMRT sequencing or nanopore approaches [121–123] may over time become alternatives to the indirect approaches currently used.

### Biases and verification

All genome-wide assays studying DNA methylation have inherent biases. Some are designed intentionally, such as the choice of oligonucleotides in microarrays, or the choice of restriction enzymes in other assays, with RRBS intentionally using short MspI fragments to target CG-dense regions, for example. Affinity-based pulldown approaches have long been appreciated to have dependence on the density of potential targets in the genome [124–126]. Even the gold standard approach of bisulphite conversion is associated with a bias involving strand specificity of sequence reads [127, 128], leading to the expectation that all of the newer assays for other cytosine variants will eventually be found to have their own systematic sources of error.

It is therefore essential to verify results obtained using genome-wide assays with more quantitative, targeted studies of individual loci, if possible using orthogonal assays. For DNA methylation, a genome-wide assay using microarrays or a restriction enzyme-based MPS assay should be tested using bisulphite sequencing at a number of individual loci, together representing the range of values obtained in the genome-wide approach, with a focus on any DMRs observed. Assays that allow relative single nucleotide polymorphism (SNP) proportions to be measured such as pyrosequencing (Qiagen) or MassArray (Sequenom) would be suitable means of testing amplicons from bisulphite-converted DNA.

Some genome-wide DNA methylation assays do not test individual nucleotides but are instead dependent upon the DNA methylation state of multiple cytosines in a region. Affinity-based assays are the best known example of this kind of dependence, but low coverage bisulphite sequencing followed by the *BSmooth* analytical approach [129] or the output of *Bumphunter* [130] are other examples. If the assay is regionally-based instead of nucleotide-based, the verification should test the cytosines throughout the region implicated to have distinctive DNA methylation, as a single cytosine may not fully represent the DNA methylation of the locus as a whole.

If SNPs (or other genomic sequence variants) are not detected in the genome-wide assay, especially if a DMR is located at a site of a known SNP, it becomes important to test whether the results reflect the presence of a sequence variant. SNPs causing effects on DNA methylation assays can be categorized into two groups: those immediately at the cytosine being tested, and those located at a distance that can affect DNA methylation at the site tested, so-called methylation quantitative trait loci (mQTLs) [131]. While the former category is relatively straightforward to identify, mQTLs can be located tens of kilobases from the site at which they affect DNA methylation [132, 133], which creates a major challenge in trying to understand whether differential DNA methylation is due to DNA sequence variability or an independent epigenetic event. We have shown that SNP genotyping to identify ancestral haplotypes can allow us to account for the effect of ancestry upon DNA methylation variability [92], which is a relatively cost-effective strategy, but our expectation is that DNA methylation studies will need to include genomic sequencing in parallel if we are to account fully for mQTL influences, which have been estimated to account for 22 to 80% of variability on DNA methylation between individuals [132, 133].

### Human disease studies and *cis*-regulatory regions

Human disease studies have to be designed with the assumption that the DNA methylation changes will be modest, in a group of individuals who may be heterogeneous in their epigenetic associations with the phenotype, and subject to confounding variables such as cell subpopulation variability and mQTLs influencing results. To design this kind of study properly requires reduction of the effects of known confounding variables to the greatest extent possible, but will also probably require cohorts of sizes larger than studied to date [105].

This impacts the choice of DNA methylation assay. Ideally, we would use the most comprehensive and quantitative assay available, WGBS. If the cell type were found through a global assay to contain reasonably substantial amounts of 5hmC, adding this information would help to discriminate the 5mC and 5hmC contributions to specific loci. Unfortunately, the cost associated with WGBS on its own is currently at least several thousand US dollars, and, as mentioned above, the depth of sequencing required for quantification of the less abundant 5hmC modification would need to be even greater, with associated costs.

It is therefore understandable that researchers have opted to use survey approaches in human disease studies. Important factors in the use of these assays for human disease studies should include ease of use, as the assay may need to be used repeatedly as samples are acquired over time, so a reproducible workflow is essential. The assay should be able to use limited sample quantities, which is often an issue with material from clinical sources, especially if cell purification is performed. Microbial contamination is inherent to certain types of epithelial samples, which would represent a potential problem in shotgun sequencing-based approaches.

A major current concern is that the survey approaches should be testing the most informative regions of the genome. The assumption to date has been that the targeting of promoters and CpG islands optimizes the information available from these assays, but as described earlier, the dynamic changes in DNA methylation that are associated with transcriptional changes appear to occur more frequently at nonpromoter *cis*-regulatory elements [42, 65–71] and at CpG island shores [63], where enhancers appear to be enriched [64]. Adding in CpG islands allows the further identification of DNA methylation acquisition at these elements in the more extreme epigenetic dysregulation occurring in cancer [55].

Human diseases are increasingly recognized to be associated with DNA methylation changes at *cis*-regulatory loci other than promoters. This is now being identified in cancer [65, 68–70] and noncancerous conditions [66, 67] as well as normal cell differentiation [71]. An obvious question that arises is whether current, commonly used DNA methylation assays represent these nonpromoter *cis*-regulatory elements adequately. Mapping *cis*-regulatory loci has been facilitated by ChIP-seq assays, with the ENCODE project using combinations of mapped chromatin states to define different functional properties of the genome, including enhancer and insulator functions [134]. To test how well several assay types represent different genomic properties, we used published WGBS and RRBS data [112], the list of loci represented by the Illumina Infinium HumanMethylation450 microarray [110] and the HpaII loci represented by the HELP-tagging assay [108]. We used the features generated by ChromHMM [135] and Segway [136] analyses of human embryonic stem cells available from the ENCODE group [137] as a source of annotations of functional elements in a reference cell type. We then measured the proportion of loci in each type of annotation overlapped by one or more loci interrogated by an assay. For example, if a type of feature is predicted by ChromHMM to occur in the genome 100 times, and the Illumina 450 K microarray has one or more probes at 50 of these genomic locations, the feature would be said to be represented 50% of the time by the microarray assay.

**Figure 3:**
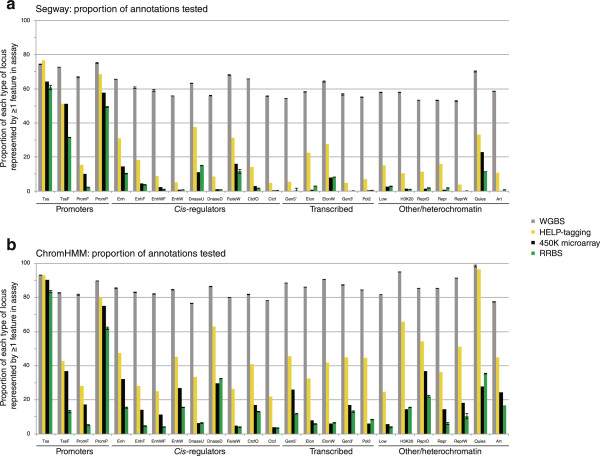
**The proportional representation of functional genomic elements by different DNA methylation assays.** Using annotations of human embryonic stem cells by Segway **(a)** and ChromHMM **(b)**, we calculated how four types of genome-wide DNA methylation assays represent each type of functional element. When the proportion of functional genomic elements tested by each assay is calculated, all of the assays work best to represent the candidate promoters annotated by Segway **(a)** or ChromHMM **(b)**, but generally only represent a minority of candidate *cis*-regulatory elements. Even WGBS does not represent 100% of each functional genomic element, so no assay achieves complete comprehensiveness, and the penalty of the WGBS approach is that it sequences equally deeply a number of genomic contexts that are not as likely to be informative, an inefficient use of costly sequencing resources.

We show the results of this analysis in Figure 3. As can be seen, WGBS is the best at representing *cis*-regulatory sequences (promoters and enhancers), with approximately 55 to 90% of these individual features represented, but also includes substantial representation at the less informative transcribed and heterochromatic loci. All of the survey assays are at their best representing promoters, but the assays all have in common that they only report a minority of the candidate *cis*-regulatory sequences predicted by Segway and ChromHMM. These results have to be interpreted with appropriate caution - deeper sequencing would increase the representation by WGBS and RRBS, as should the use of the enhanced RRBS technique [138], but it is not expected that any such measure will address the fundamental problem that the majority of the loci that are most likely to be informative are not tested by anything except the costly WGBS approach. Furthermore, as enhancers are highly cell type-specific [139], no single design for a survey assay is likely to be informative across all cell types tested in human disease studies.

### Assays targeting *cis*-regulatory regions

**Figure 4:**
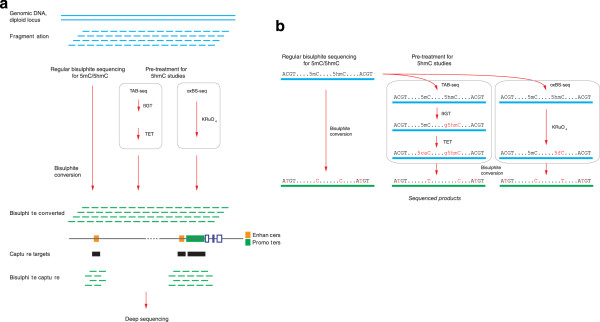
**Assays for cytosine modifications and a targeted sequencing strategy.** We show regular bisulphite sequencing, which reports both 5mC and 5hmC, and two assays detecting 5hmC alone, as examples of the broader group of assays detecting cytosine variants. Any system that captures bisulphite converted DNA can be used downstream of these sample preparation approaches to target deep sequencing to loci of interest, here indicated by *cis*-regulatory elements **(a)**. In **(b)** we show the steps and the nucleotide conversions involved in bisulphite sequencing, TAB-seq and oxBS-seq.

The focus therefore turns to how we can perform targeted bisulphite sequencing. This can be performed using multiplexed PCR for relatively limited representations of the genome [140] or padlock probes [141]. For more extensive genomic coverage, two types of capture-based assays have been described, those involving capturing DNA at target regions and then converting it using bisulphite treatment [142], and the opposite, converting with bisulphite and then capturing [143], proceeding to sequencing the enriched subset of the genome in both cases. The former approach is commercialized by Agilent as MethylSeq, the latter by Roche-NimbleGen as SeqCap Epi. Each should allow a targeting of loci in a cell type-specific manner, using enhancer predictions from the ENCODE or Roadmap in Epigenomics programs or from an investigator’s own ChIP-seq characterization of that cell type. As the enhancer landscape is much more variable between cell types than for promoters [139], a necessary component of this approach is the development of cell type-specific targeting design. The observation that hypomethylation of DNA at *cis*-regulatory loci can expand and contract suggests that the most informative sites within these loci may be those at the edges of the individual *cis*-regulatory locus [4]. With capture involved, any DNA from microbial contamination should be depleted, making it suitable for a broader range of human specimens. These capture approaches involve bisulphite sequencing, allowing the qualitative advantages of bisulphite reads to be exploited, including SNP detection, DNA methylation entropy information, and non-CG methylation. It is also reasonable to assume that a capture approach that works for bisulphite-converted DNA should also be adaptable to the assays that look for other cytosine variants (Figure 4), allowing deep sequencing and more accurate and sensitive detection of these variants as a result. At present, the most promising survey approach for DNA methylation studies in human disease would appear to be a capture-based system targeting *cis*-regulatory loci in the cell type of interest.

## Conclusions

DNA methylation has been studied for decades but it still only slowly revealing its normal physiological roles and its patterns of associations with human diseases and other phenotypes. Studies of DNA methylation remain the foundation for human disease studies, revealing not only genuine epigenetic associations but also insights into cell subtype and DNA polymorphism differences characterizing the individuals with diseases. We are moving increasingly towards the adoption of bisulphite sequencing-based approaches to interrogate DNA methylation, but are beginning to appreciate that we may need to enrich the assay’s representation of nonpromoter *cis*-regulatory sequences. As these *cis*-regulatory sites will differ substantially between cell types, we have to reconsider the idea that a single assay design will be able to serve all studies, and that instead we need to develop cell type-specific assay designs. At present, the capture-based assays appear to be best positioned to allow this kind of targeted bisulphite sequencing, with the potential for these assays also allowing targeted studies of cytosine variants other than 5mC.

### Methods

#### Sources of data used in the analyses shown

RRBS (4 Replicates):

http://genboree.org/EdaccData/Release-9/sample-experiment/H1_Cell_Line/Reduced_Representation_Bisulfite-Seq/

WGBS (2 Replicates):

http://neomorph.salk.edu/human_methylome/data.html

Methyl450K manifest:

http://supportres.illumina.com/documents/downloads/productfiles/humanmethylation450/humanmethylation450_15017482_v1-2.csv

HELP-tagging:

### Annotated HpaII sites in hg19 assembly

ChromHMM/Segway annotations of H1 ES cells:

http://ftp.ebi.ac.uk/pub/databases/ensembl/encode/integration_data_jan2011/byDataType/segmentations/jan2011/ (available as Guest login).

Description of how annotations were generated:

http://www.ncbi.nlm.nih.gov/pmc/articles/PMC3553955/bin/supp_41_2_827_v2_index.html

## Authors’ information

NU is a graduate student in the Sue Golding PhD program at the Albert Einstein College of Medicine. JMG is the director of the Center for Epigenomics and Chief of the Division of Computational Genetics of the Department of Genetics at the Albert Einstein College of Medicine and is also a clinical genomics attending physician at the Children’s Hospital at Montefiore.

## Abbreviations

5hmC: 5-hydroxymethylcytosine
5caC: 5-carboxylcytosine
5fC: 5-formylcytosine
CG (CpG): a cytosine followed by a guanine nucleotide
CHG: a cytosine followed by a non-guanine nucleotide followed by a guanine
CHH: a cytosine followed by two non-guanine nucleotides
ChIP-seq: chromatin immunoprecipitation followed by MPS
CIMP: CpG island methylator phenotype
COBRA: combined bisulphite restriction analysis
CXXC: A protein domain that binds to unmethylated CG dinucleotides
DMRs: differentially-methylated regions
DNA: deoxyribonucleic acid
DNMT: DNA methyltransferase
ENCODE: Encyclopedia of DNA elements
(G + C): The proportion of guanine and cytosine mononucleotides
H3K4me3: trimethylation of lysine 4 in histone H3
HSPCs: hematopoietic stem and progenitor cells
LINE: long interspersed nuclear element
LUMA: luminometric methylation assay
MBD: methyl-binding domain
MECP2: methylCpG-binding protein 2
MPS: massively parallel sequencing
mQTLs: methylation quantitative trait loci
NMI: non-methylated islands
PCR: polymerase chain reaction
RE: restriction enzyme
RRBS: reduced representation bisulphite sequencing
SINE: short interspersed nuclear element
SNP: single nucleotide polymorphism
TET: ten-eleven translocase enyzme
WGBS: whole-genome bisulphite sequencing.
